# Supplementary material for: Electricity generation from digitally printed cyanobacteria
Source: Nat Commun. 2017 Nov 6;8:1327. doi: 10.1038/s41467-017-01084-4 (PMC5673893; doi:10.1038/s41467-017-01084-4)
Supplement: Supplementary file 1 — Supplementary Information [file 41467_2017_1084_MOESM1_ESM.pdf]

Supplementary Figure 1

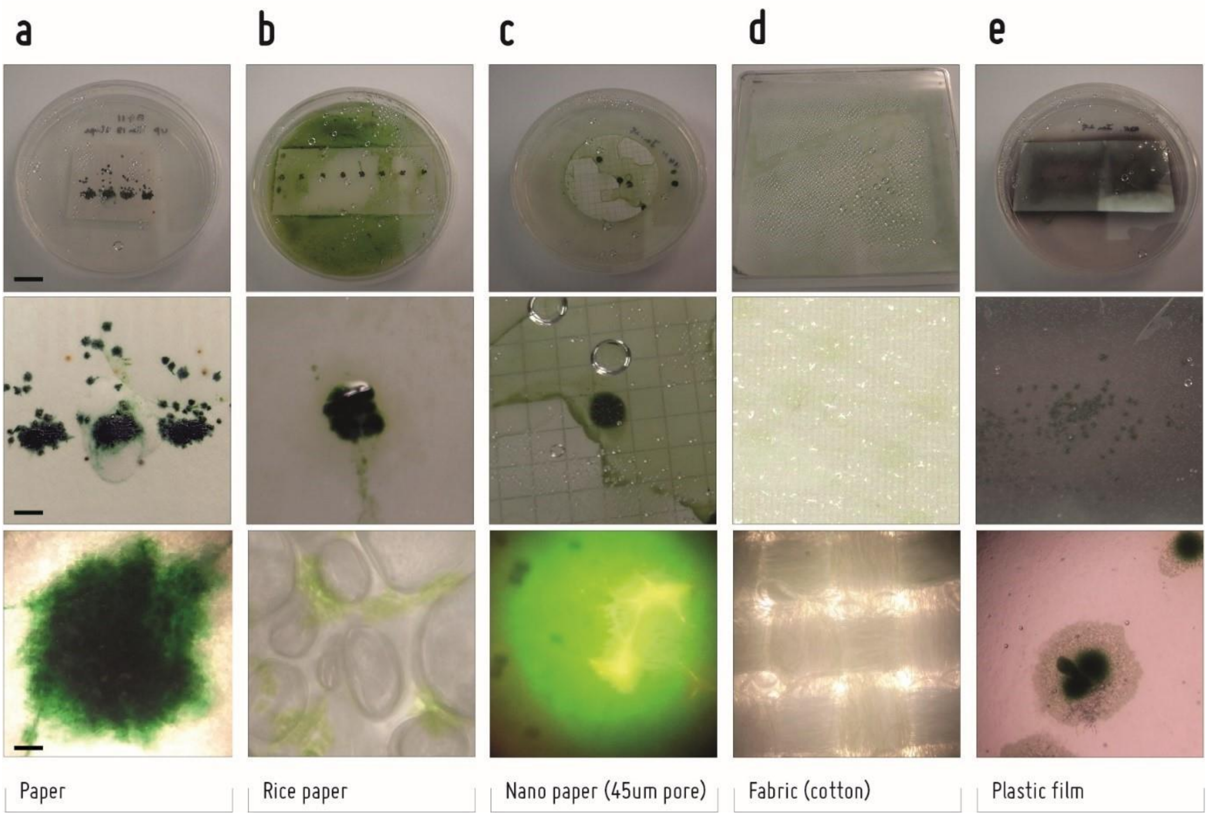

Testing biofilm growth of printed *Synechocystis* on various materials.

Photographic comparison of the growth of inkjet-printed *Synechocystis* (WT-G) on various inkjet-printable sheet substrates following 14 days of incubation: Copy paper (a), rice paper (b), nano paper (c), cotton fabric (d), and inkjet plastic film (e). There are three scale bars, one per row: from top, each measures 1 cm, 1 mm, and 100 μm respectively.

Supplementary Figure 2

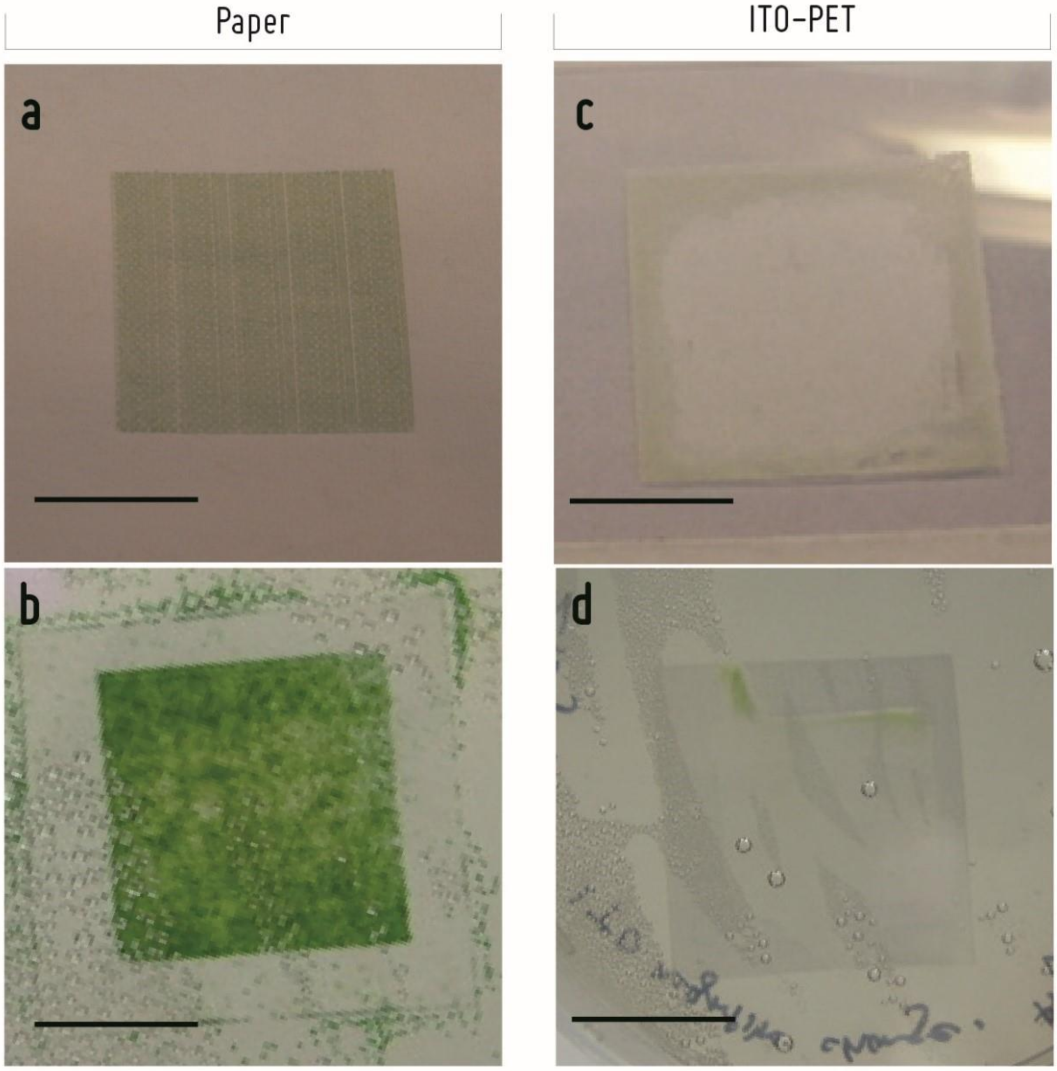

Comparison of the growth of printed *Synechocystis* cells on copy paper (a, b) and ITO-PET (c, d). Panels a and c show freshly printed cells and panels b and d the growth after 7 days of incubation on solid medium (BG-11, agar 1.5 % w/w). Each scale bar measures 1 cm.

Supplementary Figure 3

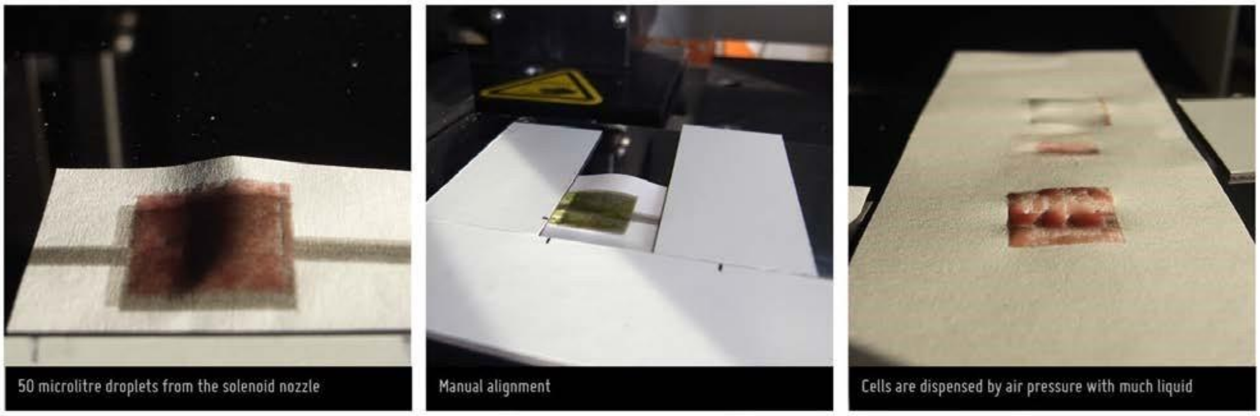

Inkjet printing of cyanobacteria using a research-grade microvalve-based cell printer. The cyanobacterium *Thermosynechococcus elongatus* BP-1 and red-pigmented cyanobacterium *Phormidium persicinum* PML 715 were printed using a Celljet (Cronus Technologies, UK). The pattern was programmed in the custom software to a basic 20 x 20 mm square. The far-left panel shows a *Phormidium* cell suspension dispensed onto paper from the solenoid nozzle with a droplet size 50 nanolitre, far larger than the 140 picolitre droplet size used in inkjet printing exploited in the present research; the middle panel - the manual alignment setup using *T. elongatus*; the far-right panel – dispensed *Phormidium* cell suspension on paper.

Supplementary Figure 4

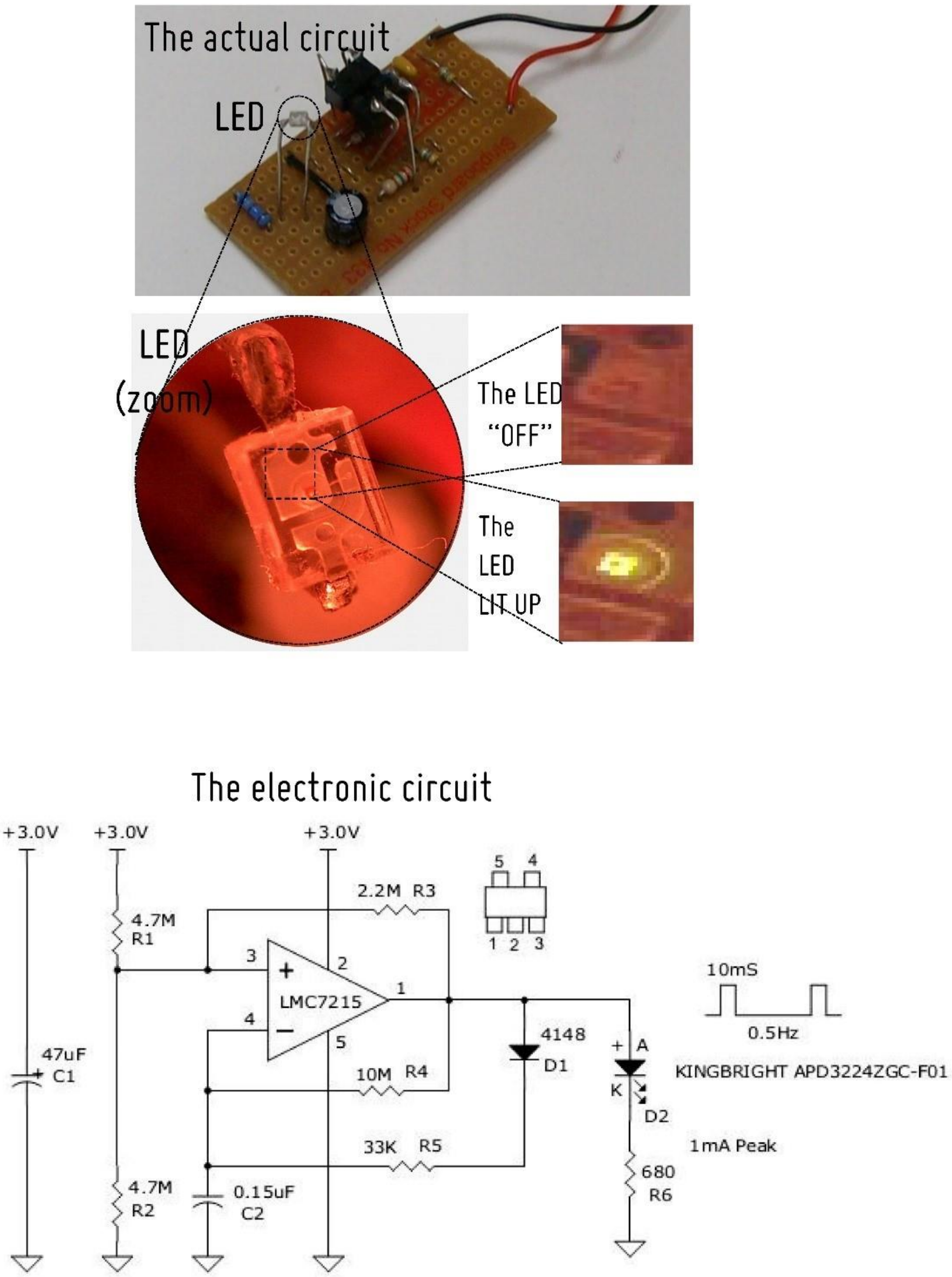

LED circuit. Photograph of the circuit (upper panel) and the electronic circuit diagram (lower panel).

Supplementary Figure 5

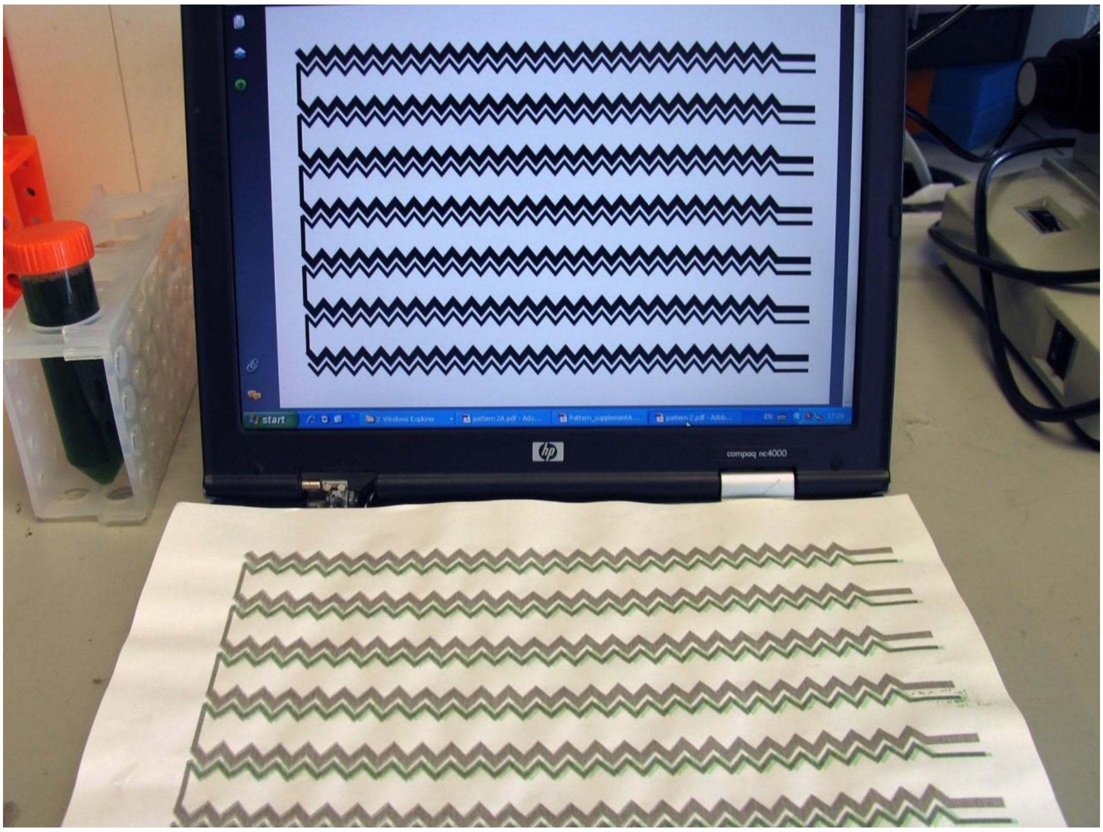

Printing of an A4-size fully printed BPV cell for bioenergy wall paper.

The panel shows a freshly printed A4-size BPV ‘paper’ containing zigzag electrodes in series, shown together with the computer-aided design file on the computer screen. Green zigzag lines are anodes of printed *Synechocystis* cells on top of preprinted CNTs.

Supplementary Figure 6

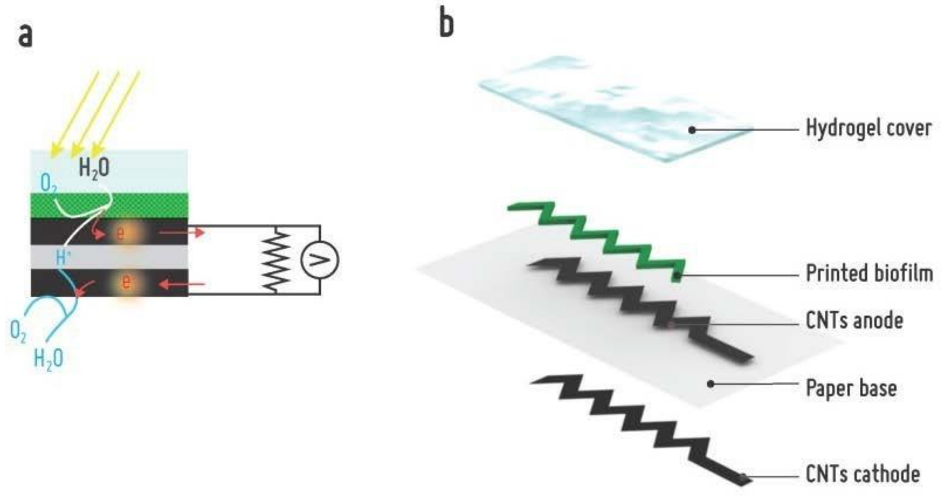

‘Sandwich’ design for the fully printed BPV device. Schematic representation of another system for the printed paper-based BPV cell in which the anode and cathode are back to back with the paper substrate in between (a). Expanded construct of the sandwich structure (b).

Supplementary Table 1

ANOVA test of the numbers of colonies grown from the most diluted cell suspension taken before printing (mean 87.5, standard deviation 12.0, n=2) versus the number of colonies grown from the most diluted cell suspension taken after printing (mean 90.5, standard deviation 10.6, n=2).

	SS	df	MS	F	p
Between:	9.000	1	9.000	0.071	0.815
Within:	254.3	2	127.2		
Total:	263.3	3			

SS: sums of squares; df: degrees of freedom; MS: mean squares; F: F-value; p: *p*-value

Supplementary Table 2

ANOVA test of the anodic peak power output produced by printed cells in the dark (mean 0.22 mW m^{-2}, standard deviation 0.07 mW m^{-2}, n=9) and printed cells in the light (0.34 mW m^{-2}, standard deviation 0.07 mW m^{-2}, n=9).

	SS	df	MS	F	p
Between:	0.115	1	0.115	23.510	<0.0005
Within:	0.078	16	0.005		
Total:	0.194	17			

SS: sums of squares; df: degrees of freedom; MS: mean squares; F: F-value; p: *p*-value

Supplementary Table 3

ANOVA test of the anodic peak power output for printed cells in the dark (mean 0.22 mW m^{-2}, standard deviation 0.07 mW m^{-2}, n=9) and the negative control (i.e., the bare printed anode) in the dark (mean 0.07 mW m^{-2}, standard deviation 0.01 mW m^{-2}, n=3).

	SS	df	MS	F	p
Between:	0.051	1	0.051	14.849	0.005
Within:	0.039	10	0.004		
Total:	0.090	11			

SS: sums of squares; df: degrees of freedom; MS: mean squares; F: F-value; p: *p*-value
